# Whole-Genome Sequences of Three Plant Growth-Promoting Rhizobacteria Isolated from Solanum tuberosum L. Rhizosphere in Tanzania

**DOI:** 10.1128/MRA.00371-20

**Published:** 2020-05-14

**Authors:** Becky N. Aloo, Ernest R. Mbega, Billy A. Makumba, Ines Friedrich, Robert Hertel, Rolf Daniel

**Affiliations:** a Nelson Mandela African Institution of Science and Technology, Department of Sustainable Agriculture and Biodiversity Conservation, Arusha, Tanzania; b University of Eldoret, Department of Biological Sciences, Eldoret, Kenya; c Moi University, Department of Biological Sciences, Eldoret, Kenya; d Georg-August University of Göttingen, Institute of Microbiology and Genetics Department of Genomic and Applied Microbiology & Göttingen Genomics Laboratory, Göttingen, Germany; University of Maryland School of Medicine

## Abstract

We present here the complete genome sequences of plant growth-promoting *Klebsiella* sp. strain MPUS7, *Serratia* sp. strain NGAS9, and *Citrobacter* sp. strain LUTT5, isolated from rhizosphere soils and tubers of potato (Solanum tuberosum L.) plants growing in the northern and southern highlands of Tanzania.

## ANNOUNCEMENT

Plant rhizospheres have long been investigated and exploited for their plant growth-promoting (PGP) rhizobacteria (1, 2). Potato tubers and rhizosphere soils were sampled from the Tanzanian northern and southern highlands for rhizobacterial isolation (3, 4). *Klebsiella* sp. strain MPUS7, *Serratia* sp. strain NGAS9, and *Citrobacter* sp. strain LUTT5, identified by partial 16S rRNA gene sequencing (5), were selected for whole-genome sequencing.

The strains were grown in Trypticase soy broth (Difco) at 37 ± 2°C for 24 h in a rotary shaker (130 rpm). Total nucleic acids were extracted with the MasterPure DNA purification kit (Epicentre, Madison, WI, USA) and used for sequence library preparations without further processing. Illumina paired-end shotgun libraries were generated with the Nextera XT DNA sample preparation kit and sequenced using the MiSeq system and reagent kit v.3 (2 × 300 bp) (Illumina, San Diego, CA, USA). For Nanopore sequencing, libraries were prepared using the ligation sequencing kit 1D (SQK-LSK108) and the native barcode expansion kit (EXP-NBD103) (Oxford Nanopore Technologies, Oxford, UK). Sequencing was performed for 72 h on a MinION Mk1B device and a SpotON flow cell R9.4 using MinKNOW software v.19.06.8 (Oxford Nanopore Technologies). The short and long reads were called with the MiSeq control software v.2.6.2.1 and Guppy v.3.2.1., respectively. Read quality assessment and processing were performed with fastp v.0.19.5 (6), resulting in 2,304,340, 2,623,096, and 2,439,948 short Illumina reads and 26,946 (*N*_50_, 19.4 kb), 34,866 (*N*_50_, 19.9 kb), and 31,389 (*N*_50_, 20 kb) long Nanopore reads for *Klebsiella* sp. MPUS7, *Serratia* sp. NGAS9, and *Citrobacter* sp. LUTT5, respectively. All kits were used as recommended by the manufacturers, and default parameters were used for all software unless otherwise specified.

Genome assemblies were performed using the Unicycler v.0.4.8 (7) pipeline with SPAdes v.3.14.0 (8), Racon v.1.4.10 (9), BLAST v.2.2.28+ (10), Bowtie 2 v.2.3.4.3 (11), SAMtools v.1.9 (12), and Pilon v.1.23 (13) and resulted three times in single circular chromosomes. The Unicycler pipeline automatically rotated all genomes, defining *dnaA* as the first protein-coding gene. The average coverage was calculated with Qualimap v.2.2.1 (14); Bowtie 2 v.2.3.4.3 (11) was used for short-read mapping, and Minimap2 v.2.17 (15) was used for long-read mapping. This resulted in 74-, 97-, and 61-fold (Illumina reads) and 121-, 216-, and 208-fold (Nanopore reads) genome mean coverage from *Klebsiella* sp. MPUS7, *Serratia* sp. NGAS9, and *Citrobacter* sp. LUTT5, respectively. BLAST analysis of the complete 16S rRNA genes of these strains showed over 99.6% similarity to Klebsiella grimontii SB73 (GenBank accession number NR_159317.1), Serratia marcescens NBRC 102204 (NR_114043.1), and Citrobacter freundii ATCC 8090 = MTCC 1658 (NR_028894.1), respectively. Gene annotation was done with the Prokaryotic Genome Annotation Pipeline v.4.8 (16).

The genome features of the strains are summarized in Table 1. Their protein-encoding genes included potassium, nitrogen, phosphorus, and iron metabolism genes, which are associated with plant growth promotion (17–19). These genomes are the first to be sequenced for potato rhizobacteria in Tanzania and can help to unravel their molecular PGP mechanisms for possible biotechnological application as biofertilizers.

**TABLE 1:** Genome features of the strains

Feature^*a*^	Value for:		
	*Klebsiella* sp. MPUS7	*Serratia* sp. NGAS9	*Citrobacter* sp. LUTT5
Genome size (bp)	5,823,634	5,155,099	5,034,577
GC content (%)	55.16	58.74	52.15
No. of genes	5,447	4,866	4,783
No. of CDS	5,331	4,736	4,661
No. of RNAs	116	130	122
No. of rRNAs	25	22	25
No. of tRNAs	84	88	84
No. of ncRNAs	7	20	13
No. of chromosomes	1	1	1
No. of coding genes	5,277	4,702	4,608
No. of pseudogenes	54	34	53

a CDS, coding DNA sequences; ncRNAs, noncoding RNAs.

### Data availability.

The whole-genome shotgun projects of *Klebsiella* sp. MPUS7, *Serratia* sp. NGAS9, and *Citrobacter* sp. LUTT5 have been deposited at GenBank under the accession numbers CP047604, CP047605, and CP047606, respectively. The versions described here are the first versions. The raw sequencing data sets of these strains have been registered in the NCBI Sequence Read Archive database (20) under the accession numbers SRP255262, SRP255259, and SRP255263, respectively.
